# Needs and skills of informal caregivers to care for a dependent person: a cross-sectional study

**DOI:** 10.1186/s12877-019-1274-0

**Published:** 2019-09-18

**Authors:** Maria dos Anjos Coelho Rodrigues Dixe, Liliana Fernanda da Conceição Teixeira, Timóteo João Teixeira Camacho Coelho Areosa, Roberta Caçador Frontini, Teresa de Jesus Almeida Peralta, Ana Isabel Fernandes Querido

**Affiliations:** 1 Center for Innovative Care and Health Technology, Escola Superior de Saúde do instituto Politécnico de Leiria, Campus 2 - Morro do Lena, Alto do Vieiro - Apartado 4137, 2411-901 Leiria, Portugal; 2 Escola Superior de Saúde do instituto Politécnico de Leiria, Campus 2 - Morro do Lena, Alto do Vieiro - Apartado 4137, 2411-901 Leiria, Portugal; 3 Centro Hospitalar de Leiria, Rua das Olhalvas, 2410-197 Leiria, Portugal

**Keywords:** Dependent person, Informal caregiver, Caregiver needs, Caregiver competencies

## Abstract

**Background:**

The world is facing many socio-demographic changes, such as an increased average life expectancy and the presence of chronic and non-communicable diseases, which in turn, leads to an enhanced dependency on others. Consequently, the demand for informal caregivers has significantly increased during the past few years. Caring for a dependent person is linked to a series of burdens that often leads to physical, psychological and emotional difficulties. Taking into consideration the difficulties faced by informal caregivers, knowing in which areas of functioning they need more guidance may help to relieve their burden. Therefore, the main goal of this study is to better understand the needs and competencies of the informal caregiver when caring for a dependent person in the different self-care domains.

**Methods:**

This cross-sectional study used a questionnaire administered on a single occasion by face-to-face interview. Descriptive and inferential statistics alongside non-parametric statistical techniques such as the Mann-Whitney test and Spearman’s correlation were used.

**Results:**

The average age of the 143 informal caregivers is 58 years old, with the youngest in our sample being 21 years of age. Most of them are female, and 50% of them are children taking care of one of their parents. Most of the dependent people are completely dependent in the areas of comfort and hygiene (53.8%) and medication management (55.9%). The female informal caregivers see themselves as having more competencies in sanitary hygiene than the male ones, with no significant differences in their competencies’ perception in the other areas of self-care. Older caregivers see themselves as less competent in certain areas of self-care such as feeding, mobility, transfers, medication and symptoms management and communication. Most of the information given to the informal caregiver is about the disease (82.3%) and the medication management (80.4%). There are still a lot of areas of self-care, where no information, or almost none, is given to the informal caregivers.

**Conclusions:**

Before home discharge of a dependent person, it is important to acknowledge the needs and competencies of the informal caregiver, to capacitate them in looking after their relatives, to help decrease their burden and consequently, decrease the number of hospital readmissions.

## Background

The world’s population is increasingly ageing in nearly every country in the world, including Portugal [1]. There has been an increase both in the average life expectancy of the population [1–3] and in the presence of chronic and non-communicable diseases [3] which is linked to the person’s survival and, often, to the presence of morbidity and dependence on others [4]. Moreover, people tend to prefer to live at home as long as possible [5]. Nonetheless, dependency cases are related to healthcare recurrences. Taking the information above into account, it is expected that the need for informal caregivers will increase in the next few years [5].

The informal caregiver usually has different responsibilities [6], providing unpaid assistance to a dependent person and performing daily life tasks concomitantly with other activities related to the healthcare of the person [7], usually related with medication, wound treatment and equipment monitoring [8]. Informal caregivers are usually family members or close relatives [9, 10] providing partial or full care to a dependent person with difficulties in self-care, facilitating their well-being and helping in different tasks and activities [11].

According to Orem [12], there are some universal self-care requirements that should be important areas of evaluation in a dependent person: 1) the maintenance of sufficient amount of air; 2) sufficient intake of water; 3) sufficient intake of food; 4) care regarding elimination processes; 5) balance between activity and rest; 6) balance between solitude and social interaction; 7) prevention of hazards to human life, the functioning, and well-being of the human being; and 8) promotion of the functioning and development of the human being within the social groups. Nonetheless, family caregivers usually report insufficient preparation for an adequate continuity of care to the dependent person on their return home [13, 14].

Moreover, and although research has found contradictory findings [15], a large percentage of studies found that caring for a dependent person is related to a series of burdens that often takes them to their physical, psychological and emotional limits [15–17], impacting the whole family system [15]. The responsibilities imposed by the caregiving situation may interrupt social life, activities or even work, which may be challenging [18]. For instance, some studies report that caregiving is usually associated with a deterioration in the quality of life [5, 6, 19, 20], higher levels of depression symptoms [5, 19, 21, 22], greater financial burden [19, 23, 24] and greater physical impairment [19, 25] especially when compared to non-carers. Therefore, caregiver burden is frequently defined as the emotional, social and financial stress imposed by the illnesses of the dependent person onto the caregiver [23, 26]. Nevertheless, informal caregivers experience challenging issues related not only to the condition of the dependent person, but also to their emotional feelings [11]. Some studies tried to better understand the reasons behind the experience of difficulties in caregivers. Pinquart and Sörensen [19], for example, explained that higher levels of psychological distress among spouses may be explained by higher levels of care provision. The caregiver burden has also been related to their own psychological distress and quality of life [26, 27].

Informal caregivers are usually aware of the difficulties they may face after the dependent person’s hospitalisation. They usually report the need for information regarding various areas of functioning, such as information regarding monitoring, management, personal care delivery, care skills, and handling emotional distress [28].

Because caregivers represent an essential part of the care network, it is important to support caregivers in managing their difficulties in caregiving [11, 29]. The quality of care provided by an informal caregiver depends on the quality of life and well-being of the caregiver itself. Moreover, and although literature refers to difficulties in caregiving, it is also acknowledged that even facing similar situations, caregivers may experience different levels of burden and/or different levels of subjective well-being [5]. It is thus of utmost importance to understand the resources these caregivers use (or will need) to minimise the physical and emotional burden they may face, which could be linked to the care provided. Health professionals may use this information to provide better care plans, enhancing supportive measures and thus, lead to more successful care conditions. Health care professionals have a set of instruments and information that might meet the specific needs of each dependent person. Consequently, promoting the well-being of caregivers and provide them with useful information should be among the main concerns of health professionals [16, 28], especially taking into account that providing support for caregivers may not be enough [15].

Besides, some of the problems faced by the caregiver at home are the major causes of readmission of the dependent person at the hospital [28]. Thus, understanding the major difficulties that the caregivers face during the provision of care is fundamental for any healthcare professional aiming to better help the caregivers to provide effective health care, which consequently may help to significantly reduce the unnecessary use of hospital services. Furthermore, and taking into consideration the difficulties faced by caregivers, knowing in which areas of functioning they need more guidance may help relieve the burden of the caregiver and, consequently, help to improve their health. Although the impact of caregiving on a dependent person has begun to be explored more extensively, there is still a lack of research regarding the specific needs and skills regarding a dependent person’s self-care. To our knowledge, there have been few studies assessing those variables all together in several areas of functioning.

Therefore, the main objectives of this study is to better understand the needs and skills of the informal caregivers of a dependent person in self-care in various areas of functioning, namely: feeding; sanitary hygiene; comfort; mobility; movement; dress and undress; medication; symptoms management and communication, to identify the degree of dependency of the dependent person; to identify the information given to the informal caregivers and to relate the skills for caring for a dependent person in self-care with gender, age and previous caregiver experience.

## Methods

### Study design

This study is reported in line with the STROBE statement [a]. This is a cross-sectional study carried out using face-to-face quantitative structured interviews based on standardized questionnaires.

### Participants and setting

The target population of this study was made up of informal family caregivers of dependent people (assessed by health care professionals using the Barthel Index) in at least one of the self-care categories and who were discharged from the medical units at a Portuguese urban hospital to their home, no matter their age, diagnosis and dependency.

The selection of caregivers was carried out by health care professionals.

Caregiver’s interviews were performed by nurses and nursing students who did not work at the unit to prevent influencing patients’ answers, following a standardised protocol.

All the interviews were carried out at the hospital at the moment of discharge home.

Participants were given information about the study and that they were free to withdraw from the study at any time for any reason, and with no obligation to give the reason for withdrawal.

The names and any other identifying details of participants were not collected in any of the surveys.

Data collection occurred from February to June 2017. In this period 900 patients were discharged from hospital, but only 324 filled in the eligibility criteria for this study. From those, 143 informal caregivers accepted to participate, representing 44,1% of caregivers of dependent people discharged home.

### Measures

The interview conducted consisted of two parts:

- Socio-demographic and family data, the experience of the caregiver,
- Caregiver’s skills specifically relating to care, professional information receive which provided them with information.

The items used to assess the caregiver’s skills in caring for a dependent person were built based on four criteria: a) the items of other instruments, b) meetings with health care professionals, c) meetings with informal caregivers, d) bibliographical research.

Given these criteria, the first version of the instrument was made up of 114 items spread over 9 domains corresponding to the areas of skills. These items have been subject to evaluation by a panel of experts using the Delphi Method. Each of these items was rated on 5 response options: Strongly Disagree; Disagree; Agree; Strongly Agree and Not applicable. For each of the areas and respective items, the informal caregiver was asked about the degree of dependence of their family member using a close-ended question with three response options: Independent, partially dependent and totally dependent.

For this study, it was pretended to access the perception of informal caregivers on the dependency of their family member. For this reason, and considering that the Barthel Index, designed to health care professionals, could not address all the issues of dependency that worries the caregivers, we decided on to create a new instrument. By using the items that caregivers referred to be relevant for their caring role, together with specific items pointed out by health care professionals, the instrument at its final version, covers different and specific areas, and it is broader than the existing instruments to measure Activities Daily Living (ADL) and Instrumental Activities of Daily Living (IADL).

After determining the internal consistency of each of the domains, the scale was composed of 101 items according to the data in Table 1. The 13 deleted items were based on two criteria: alpha of each indicator was not higher than the global alpha of their respective domains, and the results of the correlation of each item with the global rating (by removing the respective item) were not higher than 0.20. An Exploratory factor analysis was conducted and described in the analysis.

**Table 1:** Number of items in each subscale and Cronbach’s alpha value of each subscale

	N° items before validation	N° end items	Cronbach’s alpha
Feeding	24	19	0.905
Sanitary hygiene	13	10	0.787
Comfort	14	12	0.904
Mobility	12	12	0.835
Transfer	10	10	0.859
Dressing and undressing	7	6	0.685
Medication	12	10	0.808
Management of symptoms	17	17	0.911
Communication	5	5	0.544
Total items	114	101	

The factor analysis determined that 101 items were organized in the predefined domains (Additional file 1).

It should be noted that all of the domains have good reliability, except for communication domain which presents a *Cronbach’s Alpha* score lower than recommended (< 0.60). However, we have not excluded it due to its value in this area of caregivers’ skills.

### Ethical approval

The study protocol, the participant informed consent documentation were submitted to the Central Hospital of Leiria Ethics Committee (04–2017/05/02), who approved the study.

An appropriate location for data collection was always ensured, and the norms in use the Declaration of Helsinki (2014) were met.

### Analysis

Descriptive and inferential statistics were used. Taking into account the size of the sample, subsamples, and very different sample sizes, non-parametric statistical techniques were used, namely the Mann-Whitney test, Spearman’s correlation according to the type of variables under study.

The reliability of the 9 domains was measured by calculating internal consistency (Cronbach’s alpha), and individual item analysis was performed by calculating the corrected item-total correlations and α if the item was removed.

In this study means of Cronbach’s α > 0.6 are considered acceptable, α > 0.7 considered good, α > 0.8 very good and α > 0.9 are considered excellent.

Exploratory factor analysis was conducted with the 101items. Using *Varimax* rotation, and Kaiser’s eigenvalues greater than one, a nine-factor structure was extracted, explaining 61.039% of the variance. All the items correlated at least 0.3 with at least one other item, non-inclusion of items that scored in more than one factor less than 0,10 and not considering each factor with less than three items, suggested reasonable factorability. The Kaiser-Meyer-Olkin measure of 0.745, above the recommended value of 0.6 was considered good, and Bartlett’s test of sphericity (16,171.631; *p* = 0.000) indicated a good fit of the structure. The diagonals of the anti-image correlation matrix were also all over 0.5 Finally, the communalities were all above 0.392 and below 0.817, further confirming that each item shared some common variance with other items. Given these overall indicators, factor analysis was deemed to be suitable.

It should be noted that all of the domains have good reliability, with the exception of communication domain which presents a *Cronbach’s Alpha* score lower than recommended (<.60).

## Results

- **Characterisation of the sample regarding socio-demographic characteristics, professional situation and previous experience as an informal caregiver**

The 143 participating caregivers had an average of 58.7 ± 15.4 years, the youngest being 21 years old and the eldest 88 years old. 67.8% of the caregivers lives with the patient in the same household, and 90.2% had already cared for them previously, with 75.4% having help from another person to help them care for the patient. In the area of education and training, 38.5% of the caregivers completed primary school education, 16.8% completed their compulsory high school education, 14.7% have completed sixth form education, 11.9% of the sample had completed middle school education, 11.9% had completed higher education, and 6.3% had no formal education.

Of the 90.2% of caregivers who had previously cared for the dependent person, they did so on average on 89.1 ± 50.9 of days. The caregivers were predominantly female (116, 81.1%) and regarding their relationship to the patient, 50.3% were their child, 35.7% were their spouse, 2.1% were siblings, 2.1% were grandchildren or others (9.8%).

These caregivers did not care for more than one dependent person and 75.4% have help from another person to care for their dependent relative.

- **Level of dependence of the dependent person perceived by the caregiver**

These caregivers take care of 143 dependent people in at least one of the self-care activities. The average age of the 51.7% (74) female patients and the 48.3% (69) male patients is 80.7 ± 10.1 years.

By analysing Table 2, it can be seen that, in terms of self-care, the majority of people are totally dependent on comfort, and medication self-care, and that they are less dependent in terms of feeding and mobility self-care, 5.7% (8) people were discharged to their homes with a nasogastric tube, and the same was observed for the presence of urinary catheter.

b) **Ability of the informal caregiver to take care of the dependent person in their self-care needs**

**Table 2:** Distribution of the sample’s responses about the degree of dependence of the dependent person and presence of equipment

Self-care	Independent		Partially dependent		Totally dependent	
	N°	%	N°	%	N°	%
Feeding	10	7.0	84	59.2	48	33.8
Sanitary hygiene	10	7.0	71	49.7	62	43.4
Comfort	3	2.1	63	44.1	77	53.8
Mobility	12	8.4	74	51.4	57	39.9
Transfer	24	16.8	62	43.4	57	39.9
Dressing and undressing	12	8.4	66	46.2	65	45.5
Management of medication	8	5.6	55	38.5	80	55.9
Management of symptoms	22	15.4	55	38.5	66	46.2

When comparing the various values found, we confirm that, regarding management of symptoms and mobility, the caregiver, both male and female, had a lower level of agreement in the assessment of their skills to take care of their family member. It is important to note that the differences found between the skills of the male and female caregivers do not demonstrate a statistical significance except for the area of sanitary hygiene self-care (Table 3) and women self-assessed themselves as more capable than men.

**Table 3:** Results of the application of the Mann-Whitney U test on the skills of caregivers depending on the sex of the caregiver

	Women (*n* = 116)			Men (*n* = 27)			U	p
	median	Mean	SD	median	Mean	SD		
Feeding	3.15	3.16	.386	3.15	3.10	.345	1448.000	.543
Sanitary hygiene	3.27	3.24	.331	3.22	3.12	.275	1170.500	.041
Comfort	3.55	3.47	.406	3.45	3.36	.367	1247.000	.100
Mobility	2.88	2.87	.416	2.88	2.90	.305	1536.000	.877
Transfer	3.03	3.05	.401	3.03	2.98	.268	1340.000	.242
Dressing and undressing	3.34	3.36	.405	3.35	3.33	.350	1481.000	.661
Medication	3.30	3.26	.401	3.25	3.22	.374	1474.000	.634
Management of symptoms	2.83	2.81	.479	2.81	2.82	.447	1558.500	.969
Communication	3.00	2.92	.358	2.93	3.00	.471	1477.500	.643

As caregivers become older, they perceived themselves as less capable to provide care in the following domains of self-care: feeding (rs = −.241; *p* = .004); mobility (rs = −.210; *p* = .012); transfer (rs = −.169; *p* = .044); medication (rs = −.279;.001); management of symptoms (rs = −.370; *p* = .000); communication (rs = −.288; p = .000). The fact that they have previously been caregivers did not provide them with greater capability to take care of their family member, with the exception of the area of the self-care concerning management of medication (U = 364.000; *p* > 0.01).

c) **Information received by the caregiver to assist the dependent person in their self-care**

Regarding the required information given to caregivers to help them in the self-care, we found that the percentage is higher in information given about the illness suffered (82.3%) and in the self-care of management of medication (80.4%). Importantly, a large percentage (32.9%) of caregivers did not receive any information concerning the self-care areas of bathing, followed by the self-care area of getting ready and get dressed (26.6%) and toilet use (26.4%). Also noteworthy is the higher percentage (Table 4) of the caregivers who did not receive information about the financial support (39.9%) and auxiliary equipment (28.0%).

**Table 4:** Distribution of responses by the informal caregivers regarding the necessary information they received to support self-care

Information about	Necessary information		Not enough information		None		Not applicable	
	N°	%	N°	%	N°	%	N°	%
Illness	117	81.8	23	16.1	3	2.1		
Self-care bathing	77	53.8	17	11.9	47	32.9	2	1.4
Self-care get ready and get dressed	64	44.8	26	18.2	38	26.6	15	10.5
Self-care feeding	64	44.8	15	10.5	34	23.8	30	21
Self-care toilet use	58	45.0	8	6.2	34	26.4	29	22.5
Self-care transfer	59	41.3	20	14.0	33	23.1	31	21.7
Self-care mobility	58	40.6	38	26.6	34	23.8	13	9.1
Self-care taking medication	115	80.4	17	11.9	1	.7	10	7.0
Auxiliary equipment	39	27.3	35	24.5	40	28.0	29	20.3
Community services	59	41.3	24	16.8	40	28.0	20	14.0
Financial support	31	21.7	17	11.9	57	39.9	38	26.6

Regarding the source of the information the caregiver has to help in the self-care of the dependent person, among the valid responses, we have found that nurses are the professional staff who give most information to the caregiver, except for information about the disease where the main professional providing information is the doctor. It should be noted that other caregivers were indicated as sources of information for all self-care areas particularly on community services (Table 5).

**Table 5:** Distribution of responses by the informal caregivers regarding the information given to caregivers to support self-care^a^

Information on	Doctor		Nurse		Doctor and nurse		Nurse and caregiver		Others Caregiver	
	N°	%	N°	%	N°	%	N°	%	N°	%
Illness (*n* = 140)	105	75.0	7	5.0	17	12.1	0	0.0	11	7.9
Self-care bathing (*n* = 94)	5	5.3	33	35.1	2	2.1	51	54.3	3	3.2
Self-care get ready and get dressed (*n* = 89)	7	7.9	28	31.5	1	1.1	50	56.2	3	3.4
Self-care feeding (n = 89)	4	4.5	29	32.6	1	1.1	52	58.4	3	3.4
Self-care toilet use (*n* = 78)	5	6.4	20	25.6	0	0	50	64.1	3	3.8
Self-care transfer (n = 78)	2	2.6	22	28.2	2	2.6	49	62.8	3	3.8
Self-care mobility (n = 94)	11	11.7	40	42.6	1	1.1	39	41.5	3	3.2
Self-care taking medication (*n* = 132)	18	13.6	37	28.0	28	21.2	45	34.1	4	3.0
Auxiliary equipment (*n* = 77)	10	13.0	25	32.5	0	0.0	36	46.8	6	7.8
Community services (*n* = 86)	12	14.0	24	27.9	6	7.0	3	3.5	41	47.7
Financial support (*n* = 49)	5	10.2	7	14.3	4	8.2	0	0.0	33	67.3

^a^The ‘n’ corresponds to the number of caregivers who did not indicate ‘not applicable’

## Discussion

This study aimed to investigate the needs and skills of informal caregivers in caring for a dependent person in self-care. The 143 informal caregivers are family members related to the patient [9, 10] had an average age of 58.7 years, which indicates an adult sample of working age. It is important to note that a large portion of the sample is made up of informal caregivers who are also elderly, which suggests that the ageing of the population increases the number of elderly caregivers.

As was observed in the sample, the caregivers tend to be elderly, which limits their ability to care for the dependent person, demonstrating the vulnerability inherent in the age of the caregiver in carrying out many of their tasks [30].

The previous care experience does not prove increased effectiveness in caring for the dependent person. We can observe in the results, which reveal that caregivers with previous experience have difficulties in some areas. The pre-existing difficulties, often associated to the low level of education and the high level of complexity of the problems suffered by the dependent person, result in high levels of anxiety and insecurity for the caregiver, which leads to them being afraid of performing some tasks [30, 31]. In this regard, the tasks which require manual dexterity and technique to carry them out, such as well as hygiene tasks (bed bathing), transfers and mobility, medication and feeding through a tube, and tracheal aspiration have caused great concern in the caregiver [30]. Data which are similar to those presented in this study, to the extent that a large portion of the caregivers states that these aspects are one of the critical points and create greater difficulty in caring for the dependent person. Therefore, the caregivers tend to develop multiple strategies to simplify them, not taking into consideration the criteria for the implementation of the task or its degree of complexity, which could have implications for the health of the person they care for and for the caregiver [26, 30].

On the other hand, and according to the data presented, it has been observed that there are statistical differences between caregivers who are spouses and caregivers who are children, because the spouses tend to have a smaller support network and to request less informal support [19]. In line with the literature, our findings confirm that caregivers acquire formal information mostly through the health care team, particularly the nursing staff, as they have more and closer contact when compared with other professionals [19, 32]. Despite receiving information, this is not enough for a large majority of caregivers as observed in other studies [13, 14].

In recent years, especially in European countries, there has been a prevailing feeling for a need to intervene with the informal caregiver using an interdisciplinary team, especially in people with certain diseases including Alzheimer’s disease and other dementia types, in order to reduce not only the burden carried by the informal caregiver but also to reduce existing barriers (on behalf of the Right Time Place Care Consortium et al) [33]. Therefore, health care professionals should enable informal caregivers, supporting and making small tasks easier, thus reducing their exhaustion and the incorrect measures taken by informal caregivers.

### Strengths and limitations

Certain limitations of the study must be mentioned. First, most caregivers participating in the study had previous experience in caring for dependent people. Therefore, generalizability of our results is limited as the small percentage of caregivers for the first time might had influenced the data. Second, as a cross-sectional study, it was based on the survey results. Therefore, it has limitations in providing an explicit, causal inference which should be based on the temporal relationships among the variables. Third, the study did not consider the type of training interventions provided by nurses during internment and this should be recognized in interpreting our study results. Also, this study was based on the survey results. Therefore, the range of statistics was limited to the variables and their descriptions as presented in the survey and it does not explore the skills of caregivers living in rural areas compared to urban. Methodologically, the study lacks a power analysis for the sample size determination.

Nevertheless, this study was part of a larger project aiming to develop innovative solutions to help caregivers to care for their dependent relatives. This might explain the limited number of participants readmitted to the hospital during the study.

### Implications for practice and future research

This study has several implications. The high rate of hospital readmissions and the use of Emergency Departments for non-urgent situations are related to an inadequate preparation of informal caregivers to care for their dependents at the time of discharge [34, 35]. This is a major problem facing health care system impacting in both organization and economics which should attract more attention from related health service managers and policy makers. Informal caregivers represent an important part of the health care provided to dependent people. Our study revealed a caregiver profile of working-aged adults. On the other hand, as caregivers grow older their ability to care for their relatives tends to decrease. Hence a new set of policy actions will be required to support caregivers in their caring role. It has been shown in this study that nurses are the preferred source of information. Nevertheless, the information received by caregivers was not enough to reinforce their ability to perform their caring tasks. We therefore suggest the implementation of a new different training model of caregivers in which the information provided cover the gaps identified in the present study and simultaneously empower the caregiver and users in all self-care domains. The use of several sources of information as well as the inclusion of information and communication technologies (ICT) would be considered as an upgrade of caregivers training, particularly the development digital materials (videos, website, app) addressing different caring tasks in several domains. Meanwhile, it’s essential to improve communication between hospital and homecare so that caregivers could have a better support in the transition back home with a new dependency. Moreover, the schools of health sciences should focus on interdisciplinary collaboration among several health care students regarding the development of educational and training programs to empower caregivers.

This study has implications for nursing education, practice, and research. The identification of needs of caregivers, and the skills they need to care for their dependent relatives and the sources of information they use should be considered in nursing schools’ curricula. Caregivers health literacy should be embedded in nursing programs and students should be trained to develop skills to educate and empower patient and caregiver. For nursing practice in the community, the results could serve as an inspiration for the needs to be attended in-home care nursing. Nurses in the community should be aware of the profile of the caregivers and develop new strategies of caregiver’s education that include the sources of information preferred.

Longitudinal research is needed to characterise the needs and the ability of the caregivers to perform caretaking. Research should also be conducted in health career students and teachers regarding their perceptions about health literacy and the role of health care professionals in patients and caregivers’ health literacy.

## Conclusions

This paper gives us an insight into the needs of the informal caregiver of a dependent adult. Informal caregivers are an important part of the care network, and therefore they require support in how to provide that care. The quality of the care provided depends not only on the caregiver well-being but also on the information/handover of the care provided by the health care professional. Prior to home discharge of a dependent person, it is important to acknowledge the needs and competencies of the informal caregiver, to capacitate them in looking after their relatives, in order to help decrease their burden and consequently, decrease the number of hospital readmissions that may be related to the quality of care provided at home.

## Supplementary information

**Additional file 1.** Informal caregiver’s skill assessment tool. (DOCX 50 kb)

## Data Availability

The datasets used and/or analyzed during the current study are available from the corresponding author on reasonable request.

## Abbreviations

ADL: Activities Daily Living
IADL: Instrumental Activities of Daily Living
ICT: Information and communication technologies
rs: Spearman’s correlation
U: Mann-Whitney test
