# Supplementary material for: Needs and skills of informal caregivers to care for a dependent person: a cross-sectional study
Source: BMC Geriatr. 2019 Sep 18;19:255. doi: 10.1186/s12877-019-1274-0 (PMC6749667; doi:10.1186/s12877-019-1274-0)
Supplement: Supplementary file 1 — Additional file 1. Informal caregiver’s skill assessment tool. (DOCX 50 kb) [file 12877_2019_1274_MOESM1_ESM.docx]

**Additional file 1**

**Informal caregiver’s skill assessment tool**

1. **Feeding**

| **I am capable of:** | **Totally Disagree** | **Disagree** | **Agree** | **Totally Agree** | **Not applicable** |
| --- | --- | --- | --- | --- | --- |
| Understanding when to feed the dependent person |  |  |  |  |  |
| Managing the mealtimes of the dependent person |  |  |  |  |  |
| Maintaining the established meal place of the dependent person |  |  |  |  |  |
| Using strategies that encourage food intake of the dependent person when necessary |  |  |  |  |  |
| Recognise situations that increase the water needs of the dependent person. |  |  |  |  |  |
| Detecting signs of dehydration |  |  |  |  |  |
| Perceiving if the dependent person eats solids safely |  |  |  |  |  |
| Perceiving if the dependent person drinks safely |  |  |  |  |  |
| Adapting food consistency |  |  |  |  |  |
| Feeding the dependent person |  |  |  |  |  |
| Hydrating the dependent person |  |  |  |  |  |
| Positioning the dependent person for meals |  |  |  |  |  |
| Promoting the involvement of the dependent person in dietary decisions |  |  |  |  |  |
| Promoting the participation of the dependent person in activities related with their diet |  |  |  |  |  |
| Using strategies in case of choking |  |  |  |  |  |
| Removing, washing and applying prosthesis/orthosis |  |  |  |  |  |
| Perceiving if the dependent person is tired during feeding |  |  |  |  |  |
| Ask for help, from health professionals, if needed |  |  |  |  |  |
| Ask for help, from family and friends, if needed |  |  |  |  |  |

**2. Sanitary Hygiene**

| **I am capable of:** | **Totally Disagree** | **Disagree** | **Agree** | **Totally Agree** | **Not applicable** |
| --- | --- | --- | --- | --- | --- |
| Identifying the need for the dependent person to urinate and evacuate |  |  |  |  |  |
| Detecting if the skin area of the genital and anal regions is clean and dry |  |  |  |  |  |
| Washing the genital and anal region of the dependent person |  |  |  |  |  |
| Identifying the time interval between each urination and excretion of the dependent person |  |  |  |  |  |
| Selecting the assistive devices needed for urine and bowel movements |  |  |  |  |  |
| Promoting the participation of the dependent person in decisions about evacuation method and time |  |  |  |  |  |
| Promoting the participation of the dependent person in using the toilet to urinate and evacuate |  |  |  |  |  |
| Taking care of the dependent person with diapers, pads |  |  |  |  |  |
| Ask for help, from health professionals, if needed |  |  |  |  |  |
| Ask for help, from family and friends, if needed |  |  |  |  |  |

**3. Comfort**

| **I am capable of:** | **Totally Disagree** | **Disagree** | **Agree** | **Totally Agree** | **Not applicable** |
| --- | --- | --- | --- | --- | --- |
| Managing the time schedule for dependent person hygiene |  |  |  |  |  |
| Managing the established place for dependent person hygiene |  |  |  |  |  |
| Choosing the bathing equipment of the dependent person |  |  |  |  |  |
| Bathing the dependent person in the bathroom |  |  |  |  |  |
| Combing the dependent person's hair |  |  |  |  |  |
| Brushing the dependent person's teeth |  |  |  |  |  |
| Cliping the dependent person's nails |  |  |  |  |  |
| Promoting the involvement of dependent people in personal care decisions |  |  |  |  |  |
| Encouraging the participation of the dependent in personal care activities |  |  |  |  |  |
| Perceiving the need to provide personal care for the dependent person |  |  |  |  |  |
| Ask for help, from health professionals, if needed |  |  |  |  |  |
| Ask for help, from family and friends, if needed |  |  |  |  |  |

**4. Mobility**

| **I am capable of:** | **Totally Disagree** | **Disagree** | **Agree** | **Totally Agree** | **Not applicable** |
| --- | --- | --- | --- | --- | --- |
| Changing the person’s position in bed or chair / armchair |  |  |  |  |  |
| Managing time posture change of dependent person |  |  |  |  |  |
| Identifying skin signs and symptoms (colour, temperature and texture) |  |  |  |  |  |
| Understanding the difficulties of the dependent person to walk |  |  |  |  |  |
| Identifying the equipment needed to assist with the walking |  |  |  |  |  |
| Teaching to use the equipment needed to assist with the walking |  |  |  |  |  |
| Identifying the integrity of walking aids |  |  |  |  |  |
| Identifying the most appropriate footwear |  |  |  |  |  |
| Understanding the difficulty of the dependent person to climb stairs |  |  |  |  |  |
| Identifying the appropriate equipment to aid with going up and down the stairs |  |  |  |  |  |
| Teaching the dependent person to use the appropriate equipment to assist them in climbing up and down stairs |  |  |  |  |  |
| Identifying fall hazards |  |  |  |  |  |

**5. Transfer**

| **I am capable of:** | **Totally Disagree** | **Disagree** | **Agree** | **Totally Agree** | **Not applicable** |
| --- | --- | --- | --- | --- | --- |
| Perceiving the difficulty that the dependent person has with transfers |  |  |  |  |  |
| Managing the schedule taking into account the need for transfer the dependent person safely |  |  |  |  |  |
| Managing where transfers can take place in a more secure manner |  |  |  |  |  |
| Identifying which equipment may be needed to assist with the transfer |  |  |  |  |  |
| Using the appropriate equipment for the transfers (eg board, hoist) |  |  |  |  |  |
| Perceiving the need for a transfer |  |  |  |  |  |
| Transfering the dependent person to different postures |  |  |  |  |  |
| Encouraging the dependent person to make transfers in a safely manner |  |  |  |  |  |
| Ask for help, from health professionals, if needed |  |  |  |  |  |
| Ask for help, from family and friends, if needed |  |  |  |  |  |

**6. Dressing/Undressing**

| **I am capable of:** | **Totally Disagree** | **Disagree** | **Agree** | **Totally Agree** | **Not applicable** |
| --- | --- | --- | --- | --- | --- |
| Managing the clothing and footwear of the dependent person according to the temperature, time of day and season |  |  |  |  |  |
| Choosing the assistive devices to help in dressing and putting on (shoehorn, buttonhole, among others). |  |  |  |  |  |
| Asking the health care professionals for help if necessary (recommendations and / or strategies) to deal with the person’s difficulties in getting dressed |  |  |  |  |  |
| Asking the family/friends for help if necessary (recommendations and / or strategies) to deal with the person’s difficulties in getting dressed |  |  |  |  |  |
| Promoting the involvement of the dependent person in decisions about their clothing and footwear. |  |  |  |  |  |
| Encouraging the dependent person to dress and put on their shoes. |  |  |  |  |  |

**7. Medication**

| **I am capable of:** | **Totally Disagree** | **Disagree** | **Agree** | **Totally Agree** | **Not applicable** |
| --- | --- | --- | --- | --- | --- |
| Overseeing medication intake |  |  |  |  |  |
| Detecting medication side effects |  |  |  |  |  |
| Understanding if medication is producing desired effects |  |  |  |  |  |
| Deciding what to do if any complications / medication side effects occur |  |  |  |  |  |
| Respecting medication time |  |  |  |  |  |
| Giving the correct dose of medication |  |  |  |  |  |
| Ask for help, from health professionals, if needed |  |  |  |  |  |
| Ask for help, from family and friends, if needed |  |  |  |  |  |
| Promoting the involvement of the dependent person in medication-related decisions |  |  |  |  |  |
| Encouraging the participation of the dependent person in medication preparation and intake |  |  |  |  |  |

**8. Management of symptons**

| **I am capable of:** | **Totally Disagree** | **Disagree** | **Agree** | **Totally Agree** | **Not applicable** |
| --- | --- | --- | --- | --- | --- |
| Understanding when the person is confused / disoriented |  |  |  |  |  |
| Acting in the face of confusion / disorientation of the dependent person |  |  |  |  |  |
| Realising when the person is in pain |  |  |  |  |  |
| Acting in case of pain |  |  |  |  |  |
| Realising when the dependent person is short of breath |  |  |  |  |  |
| Acting in case the person is short of breath |  |  |  |  |  |
| Realising when the dependent person has nausea/ vomiting |  |  |  |  |  |
| Acting on the nausea/vomiting of the dependent person |  |  |  |  |  |
| Realising when the dependent person has bowel changes (diarrhoea, constipation) |  |  |  |  |  |
| Acting on the bowel changes of the dependent person (diarrhoea, constipation) |  |  |  |  |  |
| Realising when the dependent person feels anxious |  |  |  |  |  |
| Acting in a anxiety episode of the dependent person |  |  |  |  |  |
| Perceive when the dependent person has a fever |  |  |  |  |  |
| Acting in a fever situation |  |  |  |  |  |
| Acting in a situation of insomnia |  |  |  |  |  |
| Acting in a situation of aggression |  |  |  |  |  |
| Acting in a situation of sadness |  |  |  |  |  |

**9. Communication**

| **I am capable of:** | **Totally Disagree** | **Disagree** | **Agree** | **Totally Agree** | **Not applicable** |
| --- | --- | --- | --- | --- | --- |
| Communicating verbally with the dependent person |  |  |  |  |  |
| Understand what the dependent person wants to convey to me |  |  |  |  |  |
| Making myself clear to the dependent person |  |  |  |  |  |
| Using equipment and/or communication support systems (tablet, notebook, computer program, among others) |  |  |  |  |  |
| Using communicative strategies (writing, drawing, gestures, among others) |  |  |  |  |  |
